# Multiple Sebaceous Cysts on the Scrotum: A Rare Surgical Occurrence

**DOI:** 10.7759/cureus.39607

**Published:** 2023-05-28

**Authors:** Vinayak V Kshirsagar, Vidita Modi

**Affiliations:** 1 Department of General Surgery, Dr. D.Y Patil Medical College, Hospital and Research Centre, Pune, IND

**Keywords:** fournier gangrene, excision, flap, epidermoid cyst, sebaceous cyst

## Abstract

A sebaceous cyst is a benign encapsulated, subepidermal nodule filled with keratin material. They are mostly seen in areas with body hair such as the scalp, face, neck back, and scrotum. Having several sebaceous cysts on the scrotum is an uncommon occurrence, and if they become infected or look unsightly, they should be removed. Histologically, cysts are lined by stratified squamous epithelium and contain keratin debris and cholesterol. If the cysts are extremely swollen or infected, the entire scrotal wall must be removed, and the testicles should be covered. This is an unusual case where the patient had multiple painless nodules of varying sizes covering almost the entire scrotal skin. These were identified as sebaceous cysts and had been present for several months. All the cysts had to be removed in toto because of such an unusual presentation which was covering the entire scrotal skin.

## Introduction

Sebaceous cysts, also known as epidermoid cysts, are common benign growths that form beneath the skin and may contain fluid or semisolid material. Sebaceous cysts form when a hair follicle's sebaceous gland becomes blocked, causing an accumulation of sebum. While they are usually painless, they can become red and painful if they become infected. These growths are frequently found in areas of the body with hair follicles, including the scrotum. While single cysts on the scrotum are relatively common, multiple cysts are rare. It is even more uncommon to have multiple cysts that cover the entire scrotal skin and are larger than 1 cm. In this case, we describe an atypical situation where a patient had several sebaceous cysts of different sizes that almost entirely covered the scrotal skin.

## Case presentation

**Figure 1:**
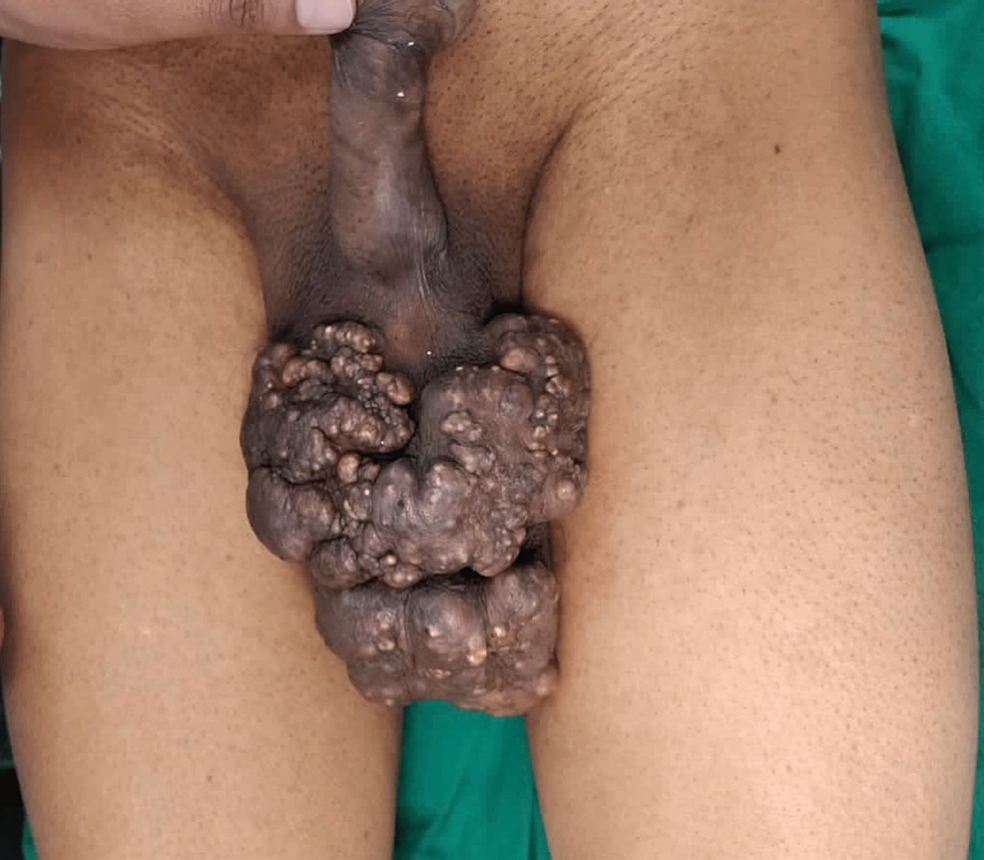
Multiple sebaceous cysts over the scrotum

A 45-year-old male presented to the hospital with chief complaints of multiple painless nodules on his scrotum that had been present for several months (Figure 1).

**Figure 2:**
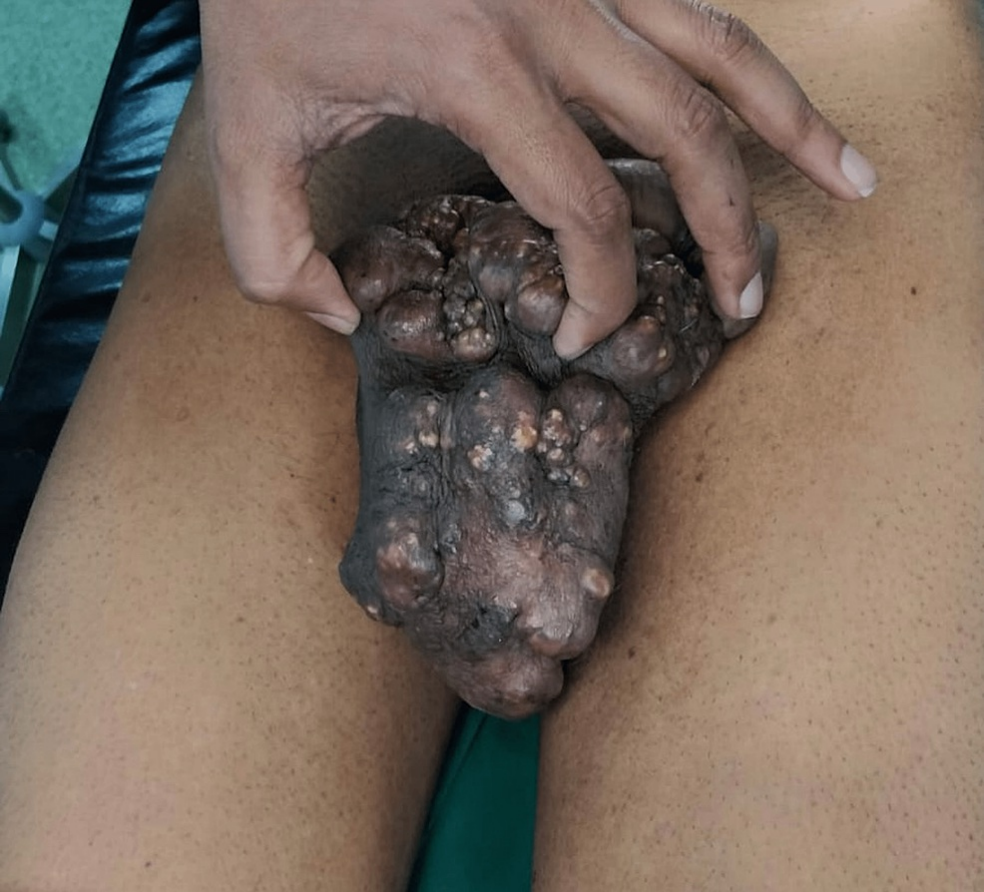
Base of the scrotum showing multiple sebaceous cysts

The nodules had gradually increased in size and number over time. The patient denied any pain, discharge, or bleeding from the nodules. There was no significant medical or family history. Upon physical examination, multiple nodules ranging in size from 0.5 to 2 cm were observed, with the largest being 2 cm. They coalesced to form a mass over the scrotum (Figure 2).

Based on the clinical presentation, a diagnosis of multiple sebaceous cysts was made. The patient was informed of the potential for infection or cosmetic concerns and was advised to have the cysts surgically removed. The patient agreed, and the surgical excision was performed under spinal anesthesia.

**Figure 3:**
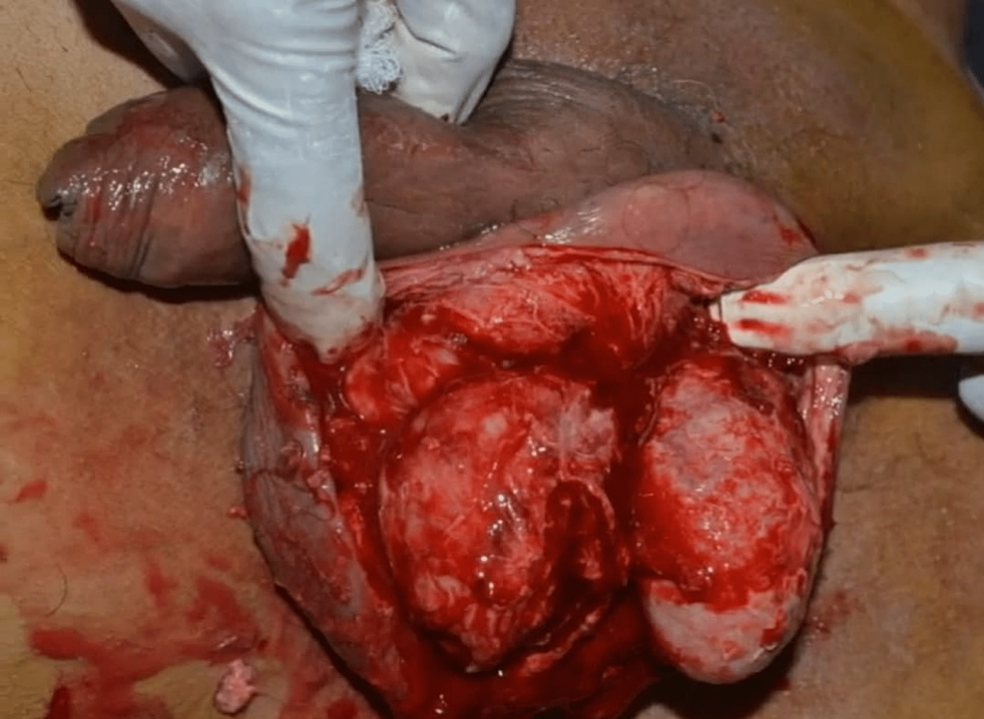
Intra-op picture post excision of all the sebaceous cysts in toto

The minimal excision technique was employed, and the cysts were removed by making an elliptical incision all around the cysts. Multiple cysts were excised in toto and sent for histopathological examination with an intra-op picture shown below (Figure 3). Flaps were raised on either side and hemostasis was achieved. A closed suction drain was placed and the scrotal wall was reconstructed by approximating the flaps. Histopathological examination of the cysts confirmed the diagnosis ruling out malignancy. The patient was started on injection amoxicillin + clavulanic acid in the postoperative period for five days.

The patient's hospital stay was uneventful and was discharged on postoperative day five. There was no recurrence of the cysts during the six-month follow-up period.

## Discussion

A sebaceous cyst is a cyst that forms in the skin and is typically small and dome-shaped [1]. It is filled with a thick, oily material that resembles cheese. Sebaceous cysts appear as a dome-shaped protrusion on the skin and are typically white or skin-colored. They can range in size from 1 to 4 cm in diameter and may occur singly or in clusters. While these cysts are usually painless, they can become inflamed, painful, and red if infected. The cause of these cysts is not entirely clear, but they often develop when a sebaceous gland's duct becomes obstructed, resulting in the accumulation of sebum and the formation of a retention cyst. Other potential causes may include a developmental defect in the sebaceous duct or the traumatic implantation of surface epithelium beneath the skin. Although sebaceous cysts can occur at any age, they are typically first noticed in adulthood.

Multiple sebaceous cysts on the scrotum are uncommon; because they are typically painless, men often tend to ignore them. However, since these cysts are located in a region that can potentially become infected, they can get infected themselves from the genitourinary tract. An infected cyst can become painful and may even rupture, leading to the discharge of pus. If a single cyst is infected, it can be drained with no problems. However, if left untreated, the infection can spread to other cysts nearby and eventually reach the scrotal wall. If the scrotum's skin is infected, the affected area must be removed widely to prevent necrotizing fasciitis (also called Fournier's gangrene) of the scrotum and sepsis.

Cannistra et al. [2] utilized the pedicle inguinal flap technique to reconstruct the scrotum in cases of Fournier gangrene. This approach enables coverage of the scrotal region with relatively thick and sensitive tissue, with minimal scarring and functional impairment. Kochakarn et al. [3] conducted a study on 12 cases in which they suggested implanting exposed testes in an upper thigh pouch, followed by delayed reconstruction of the scrotum using thigh pedicle flaps. They observed excellent outcomes using this technique. In a similar vein, Monteiro et al. [4] outlined a method for covering the testes in Fournier's gangrene cases using inner thigh flaps. Although the main complication associated with this approach is infection, flap necrosis can also occur. Nevertheless, broad-based flaps can be used to prevent flap necrosis.

Sebaceous cysts are typically benign, meaning they are not cancerous. However, there is a small chance that some of these cysts may undergo malignant changes [1]. If a patient experiences the occurrence of multiple or recurring cysts, it is important to consider the possibility of Gardner syndrome, which is an inherited condition characterized by the development of non-cancerous growths. This syndrome can give rise to growths in different parts of the body, such as fibromas, desmoid tumors, and sebaceous cysts, alongside colon polyps [2,4].

Ultrasound has emerged as the main imaging technique for examining both intra-scrotal and extra-scrotal abnormalities. When performing an ultrasound, an epidermoid cyst will exhibit distinct characteristics. It will appear as a well-defined, circular, or oval hypo-echoic lesion, meaning it appears darker than the surrounding tissue. There may be some scattered echoes within the cyst, but no signs of internal blood flow will be observed when using color Doppler imaging [5].

## Conclusions

Multiple sebaceous cysts on the scrotum are rare. These cysts can be surgically excised using the minimal excision technique. This technique is associated with low complication rates and shorter healing times, making it a suitable option for patients with multiple scrotal cysts. Early surgical intervention can prevent potential complications and provide symptomatic relief. In this particular case, we encountered a rare occurrence where the patient had multiple sebaceous cysts measuring over 1 cm in size. To address this situation, a significant portion of the scrotal skin had to be surgically removed.
